# Decreased physical activity is a predictor for a complicated recovery post cardiac surgery

**DOI:** 10.1186/s12955-016-0576-6

**Published:** 2017-01-07

**Authors:** Charlotte van Laar, Simone T. TImman, Luc Noyez

**Affiliations:** Department of Cardio-Thoracic Surgery – 615, Heart Center, Radboud University Nijmegen Medical Center, PO Box 9101, 6500 HB Nijmegen, The Netherlands

**Keywords:** Cardiac surgery, Physical activity, Morbidity, Postoperative

## Abstract

**Background:**

Physical activity (PA) reduces the risk of cardiovascular disease and physically active survivors of a cardiac event are at lower risk of recurrent events. We hypothesized that patients with a decreased PA, undergoing cardiac surgery, are at higher risk for a postoperative complicated recovery (PCR).

**Methods:**

Three thousand three hundred eighty two patients undergoing elective cardiac surgery between January 2007 and December 2013 were included. The group was divided into three subgroups: group A, aged ≤ 65 years (*N* = 1329); group B, aged > 65 years and ≤ 75 years (*N* = 1250); and group C aged >75 years (*N* = 803). To assess PA, the criteria of the Corpus Christy Heart Project were used. A PCR consists of the occurrence of a major postoperative event, defined as any of the following complications: reoperation, deep sternal wound infection, renal failure, stroke, postoperative ventilation > 2 days, intensive care stay ≥ 5 days, hospital stay ≥ 10 days, or hospital mortality.

**Results:**

One thousand three hundred sixty seven patients (40%) were considered as patients with a decreased PA. Both in group B (*p* = 0.001) and in group C (*p* = 0.003), patients with a decreased PA were significantly associated with an increased risk of a PCR, which was not the case in group A (*p* = 0.28). Logistic regression analysis identified a decreased PA as an independent predictor for PCR in groups B (*p* = 0.003, odds 1.71) and C (*p* = 0.033, odds 1.48), but not in group A (*p* = 0.11, odds 0.71).

**Conclusion:**

Decreased physical activity is an independent predictor for a PCR in patients aged 65 years or older undergoing elective cardiac surgery.

## Background

Physical activity (PA) reduces the risk of cardiovascular disease andsurvivors of a cardiac event who are physically active are at lower risk of recurrent events, especially in the elderly [1–4]. It is also known that immobility post cardiac surgery contributes to an increased risk of postoperative complications and an increased hospital stay [5, 6]. However, not much is known about the influence of the preoperative PA status on the postoperative outcome after cardiac surgery. It is presupposed that patients with a decreased PA are at higher risk for a postoperative complicated recovery (PCR), but there is no evidence that decreased PA is a predictor for PCR in a ‘normal’ patients population undergoing cardiac surgery.

The aim of the current study is to evaluate whether or not a decreased PA is an independent preoperative risk factor for in hospital morbidity and mortality post elective cardiac surgery. It is hypothesized that patients with a decreased PA undergoing cardiac surgery are at higher risk for PCR than active patients.

## Methods

### Patients and study design

This study is a single-center retrospective cohort study performed at the Department of Cardio-thoracic Surgery, Radboud University Nijmegen Medical Center at Nijmegen, the Netherlands. All patients were informed preoperatively and participated in this PA research voluntarily. Registration of data in the Cardiac Surgery Database Radboud Hospital (CORRAD) and the use of this information for research have been approved by the local ethical and research councils of the Radboud University, Nijmegen [7].

From our CORRAD database – a database that prospectively stores pre-, per-, and post-operative data plus follow-up data from all adult patients undergoing cardiac surgery at the Radboud University Nijmegen Medical Centre – we identified 5091 consecutive patients who underwent isolated CABG, isolated aortic valve replacement (AVR) or combined AVR with CABG between January 2007 and December 2013. 4544 of these 5091 surgeries (89,2%) were elective and 3382 (75%) of those elective patients completed the preoperative questionnaire concerning PA. Of the remaining 3382 patients who constituted our study group, 2484 patients underwent an isolated CABG, 523 patients an isolated aortic valve replacement (AVR) and 372 patients a combined AVR + CABG. It must be clear that non-elective surgical procedures, as well as elective patients without preoperative PA-information (a total of 1162 patients) were excluded from this study. We divided the study population into three subgroups: group A included young patients aged ≤ 65 years (*N* = 1329); group B, middle aged patients aged > 65- ≤ 75 years (*N* = 1250); and group C elderly patients aged > 75 years (*N* = 803). The reason for this split is that, in a previous report, a sedentary lifestyle couldn’t be identified as an independent predictor for hospital and early mortally, which raised the suggestion to distinguish between different age groups [8]. The logistic EuroSCORE was used for risk stratification [9].

### Physical activity

To assess the physical activity (PA), the criteria of The Corpus Christi Heart Project (CCHP) were used [3]. The activity levels are coded from 1 to 5, with 1 being sedentary and 5 being vigorously active. Details regarding the five different activity levels are summarized in Table 1. As in CCHP, the patients with level 3, 4 and 5 were collapsed into a single active group, while the other two were combined into a single sedentary group [3]. We considered this sedentary group as patients with a decreased physical activity.

**Table 1:** Categorization of physical activity, patients, and definitions

2 activity levels	5 activity levels	Patients	Activity description	Activity examples
Sedentary	Level 1 Sedentary	331	Essential no PA above minimum demands of daily living	Watching TV, working at desk, riding car
	Level 2 Minimally	1036	Activity during normal daily routine, 15–30 min/d, very light to fairly light exertion	Some stair-climbing, light gardening, light housekeeping, light home repairs.
Active	Level 3 Mildly	1257	Activity to exercise muscle groups, 15–30 min/d, fairly light to somewhat hard exertion	Calisthenics, lifting weights, heavy gardening, heavy housekeeping
	Level 4 Moderately	490	≥1 dynamic activities performed 1-3 times/week, 15 min/session, marked increase in heart rate or somewhat hard exertion	Running, jogging, bicycling, fast walking, dancing, tennis,
	Level 5 Vigorously	268	≥ 1 dynamic activities performed 3 times/week, 20 min/session, somewhat hard to hard exertion	Vigorous calisthenics, aerobic dancing, aerobic workouts, competition sport

### Outcome measure and studied risk factors

The endpoint of this study is a PCR, defined as any of the following postoperative complications or major adverse events (MAEs): reoperation for any cause, deep sternal wound infection, renal failure as defined by the Society of Thoracic Surgeons (STS) [10], stroke as permanent stroke with a permanent deficit and a transient ischemic attack (post CVA/TIA), ventilation >2 days, Intensive Care Unit stay (ICU stay) ≥ 5 days, hospital stay ≥ 10 days, or hospital mortality, occurring at any time during initial hospital admission in the cardiac surgery centre. These different variables of PCR were indicated as MAE-variables. The variables ICU-stay ≥ 5 days and hospital stay ≥ 10 days were included as MAE’s because of their occurrence in about 5% of the included patients - mostly due to one of the MAE’s, but in some cases also as a consequence of other complications such as postoperative infections or delirium, resulting in a longer ICU- or hospital stay.

The studied risk factors for PCR were: female gender, age, a body mass index (BMI) >30, insulin dependent diabetes, a history of neurological event defined as a TIA or CVA preoperatively, extracardiac arteriopathy, renal dysfunction, chronic pulmonary disease, recent infarction and previous cardiac surgery. The latter five variables are defined in accordance with the EuroSCORE [9].

### Statistical analyses

Statistical analyses were performed using IBM SPSS statistics 20, Chicago, IL, USA. Baseline characteristics are presented as percentage for dichotomous variables, as mean ± standard deviation (S.D.), or as a range for numerical variables. Differences in baseline characteristics were assessed using the Chi-square test or the Fisher exact test for discrete variables and the 2-sample students *t*-test for continuous variables. A decreased PA is studied as independent risk factor for PCR using binary logistic regression. A *P*-value of ≤ 0.05 is considered significant.

## Results

### Patients

The distribution of the total study group over the five different PA levels is presented in Table 1. 59.8% (*N* = 2015) were scored as active (level 3, 4 and 5), while 40.2% (*N* = 1367) were scored as sedentary and thus considered as patients with a decreased PA.

With respect to age, no statistically significant difference was found between the elective patients excluded because of lack of PA information (67.32 ± 10.2 (27–87) years) and those included (67.21 ± 9.7 (19–93) years). However, patients without PA information had EuroSCORE values that were significantly higher than those of the patients included (4.37 ± 4.4 versus 3.88 ± 3.9, (*p* = 0.001)), as well as more MAE’s (19% (220/1162) versus 13% (444/3382) (*p* = 0.001)).

### Hospital morbidity and mortality

Table 2 presents the incidence of a PCR and MAE-variables for the three age groups, subdivided into active patients and patients with a decreased PA. The percentage of PCR increases with age (*p* = 0.008), from 8.7% in group A to 12.5% in group B and 20.9% in group C. Groups B and C also show a statistically significantly higher percentage of MAE’s in patients with a decreased PA (*p* = 0.001 and *p* = 0.003, respectively). In group A, however, there was a higher percentage of MAE’s in the active group, although this score was not statistically significant. No significant differences were detected with respect to the MAE-variables reoperation, post CVA/TIA, or hospital mortality, in either of the 3 different subgroups.

**Table 2:** Incidence of a PCR and different MAE-variables by age and physical activity status

	Group A			Group B			Group C		
	Age ≤ 65 years			Age >65 and ≤75			Age >75 years		
	*N* = 1329			*N* = 1250			*N* = 803		
Variable	Active	DPA	*p*-value	Active	DPA	*p*-value	Active	DPA	*p*-value
	*N* = 832 (%)	*N* = 497 (%)		*N* = 777 (%)	*N* = 473 (%)		*N* = 406 (%)	*N* = 397 (%)	
PCR	78 (9.4)	37 (7.4)	0.280	76 (9.8)	84 (17.8)	0.000	68 (16.7)	97 (24.4)	0.003
Reoperation	43 (5.2)	13 (2.6)	0.025	39 (5.0)	30 (6.4)	0.319	35 (8.6)	35 (8.8)	0.922
Deep sternal wound infection	1 (0.1)	2 (0.4)	0.560	1 (0.1)	9 (1.9)	0.001	2 (0.5)	2 (0.5)	1.000
Post-CVA/TIA	3 (0.4)	4 (0.8)	0.435	2 (0.3)	5 (1.1)	0.112	3 (0.7)	4 (1.0)	0.723
Renal failure	7 (0.8)	6 (1.2)	0.512	14 (1.8)	17 (3.6)	0.048	11 (2.7)	17 (4.3)	0.225
Ventilation > 2 d	5 (0.6)	5 (1.0)	0.515	6 (0.8)	11 (2.3)	0.021	6 (1.5)	12 (3.0)	0.139
ICU-stay ≥5 d	5 (0.6)	4 (0.8)	0.735	6 (0.8)	15 (3.2)	0.001	4 (1.0)	21 (5.3)	0.000
Hospital Stay ≥10 d	22 (2.6)	13 (2.6)	0.975	18 (2.3)	38 (8.0)	0.000	16 (3.9)	51 (12.8)	0.000
Hospital mortality	0	2 (0.4)	0.140	1 (0.1)	4 (0.8)	0.071	4 (1.0)	8 (2.0)	0.229

*DPA* decreased Physical Activity, *PCR* postoperative complicated recovery, *ICU* intensive care unit

In group A no significant difference in the various MAE-variables was observed between active patients and those with a decreased PA, with exception of a significant higher percentage of reoperations in the active group (*p* = 0.025) The patients with a decreased PA of group B, however, are at higher risk for deep sternal wound infections (*p* = 0.001), renal failure (*p* = 0.048), ventilation > 2 days (*p* = 0.021), ICU stay ≥ 5 days (*p* = 0.001) and hospital stay ≥ 10 days. In group C, these patients had a higher risk of a prolonged ICU stay (*p* = 0.001) and hospital stay ≥ 10 days (*p* = 0.001).

### Risk factors associated with a decreased physical activity

Table 3 presents different well-known risk factors for mortality and morbidity for the three age groups. In group A, the following risk factors were associated with a decreased PA: female gender (*p* = 0.001), BMI > 30 (*p* = 0.001), insulin dependent diabetes (*p* = 0.001), extracardiac arteriopathy (*p* = 0.001), renal dysfunction (*p* = 0.035), chronic pulmonary disease (p = 0.004), recent myocardial infarction (*p* = 0.027) and previous cardiac surgery (*p* = 0.041). In group B, the variables female gender (*p* = 0.001), BMI > 30 (*p* = 0.001), insulin dependent diabetes (*p* = 0.003), extracardiac arteriopathy (p = 0.001), renal dysfunction (*p* = 0.001) and chronic pulmonary disease (*p* = 0.022) were significantly higher in the group of patients with a decreased PA. The patients with a decreased PA in group C, in comparison to the active patients of that group, were more often female (*p* = 0.001), had a higher BMI (*p* = 0.001), had more extracardiac arteriopathy (*p* = 0.001) and more neurological events in their history (*p* = 0.038).

**Table 3:** Risk factors for a complicated postoperative recovery by age and physical activity status

	Group A			Group B			Group C		
	Age ≤ 65 years			Age >65 and ≤75			Age >75 yrs		
	*N* = 1329			*N* = 1250			*N* = 803		
Variable^a^	Active	DPA	*p*-value	Active	DPA	*p*-value	Active	DPA	*p*-value
	*N* = 832 (%)	*N* = 497 (%)		*N* = 777 (%)	*N* = 473 (%)		*N* = 406 (%)	*N* = 397 (%)	
Female gender	117 (14.0)	118 (23.7)	0.000	155 (19.9)	159 (33.6)	0.000	114 (28.1)	193 (48.6)	0.000
BMI > 30	196 (23.6)	186 (37.4)	0.000	150 (19.3)	171 (36.2)	0.000	67 (16.5)	106 (26.7)	0.000
Insulin dependent diabetes	48 (5.8)	54 (10.9)	0.001	66 (8.5)	65 (13.7)	0.003	31 (7.6)	39 (9.8)	0.272
Extracardiac arteriopathy^a^	73 (8.8)	76 (15.3)	0.000	125 (16.1)	111 (23.5)	0.001	48 (11.8)	105 (26.4)	0.000
History of a neurological event	55 (6.6)	42 (8.5)	0.212	76 (9.8)	63 (13.3)	0.054	53 (13.1)	73 (18.4)	0.038
Renal dysfunction^a^	4 (0.5)	8 (1.6)	0.035	4 (0.5)	14 (3.0)	0.000	2 (0.5)	7 (1.8)	0.104
Chronic pulmonary disease^a^	47 (5.6)	49 (9.9)	0.004	77 (9.9)	67 (14.2)	0.022	41 (10.1)	52 (13.1)	0.184
Recent infarction^a^	87 (10.5)	34 (6.8)	0.027	56 (7.2)	26 (5.5)	0.236	36 (8.9)	33 (8.3)	0.779
Previous cardiac surgery^a^	20 (2.4)	22 (4.4)	0.041	25 (3.2)	19 (4.0)	0.457	10 (2.5)	12 (3.0)	0.627

For variables marked with ^a^ the definitions as in the logistic EuroSCORE are used. DPA = decreased physical activity, BMI = Body Mass Index, history of a neurological event = a CVA or TIA preoperative

### Predictors of a postoperative complicated recovery (PCR)

Table 4 presents the results of our binary logistic regression analysis. A decreased PA could not be identified as an independent predictor for PCR in group A. A history of a neurological event was the only independent predictor for a PCR in these young patients (*p* = 0.016, odds 2.08). In group B, both renal dysfunction (*p* = 0.001, odds 6.6) and a decreased PA (*p* = 0.003, odds 1.71) are identified as independent predictors for a PCR. In group C, a decreased PA (*p* = 0.033. odds 1.48), insulin dependent diabetes (*p* = 0.015, odds 1.97), and extracardiac arteriopathy (*p* = 0.049, odds 1.52) are identified as independent predictors for a PCR.

**Table 4:** Results of binary logistic regression analysis; predictors of a complicated postoperative recovery

Variable	≤65 years *N* = 116 (8.7%)			>65 and ≤75 *N* = 160 (12.8%)			>75 years *N* = 168 (21.9%)		
	OR	95% C.I.	*p*-value	OR	95% C.I.	*p*-value	OR	95% C.I.	*p*-value
Decreased PA	0.711	0.446–1.058	0.114	1.717	1.206–2.443	0.003	1.488	1.032–2.146	0.033
BMI > 30	1.108	0.725–1.693	0.637	1.082	0.733–1.598	0.691	0.900	0.589–1.376	0.627
Insuline dependent diabetes	1.661	0.890–3.098	0.111	1.489	0.950–2.450	0.117	1.977	1.143–3.420	0.015
Extracardiac arteriopathy	0.913	0.495–1.684	0.770	1.406	0.937–2.109	0.099	1.525	1.001–2.323	0.049
History of a neurological event	2.084	1.147–3.786	0.016	1.572	0.975–2.534	0.064	0.905	0.561–1.4269	0.684
Renal dysfunction	1.869	0.388–8.996	0.435	6.633	2.491–17.664	0.001	1.460	0.340–6.262	0.611
Chronic pulmonary disease	1.581	0.825–3.031	0.167	1.202	0.729–1.983	0.470	1.218	0.727–2.041	0.453
Recent infarction	0.780	0.368–1.653	0.517	1.429	0.757–2.698	0.271	0.903	0.475–1.717	0.756
Previous cardiac surgery	1.061	0.365–3.079	0.914	0.894	0.343–2.333	0.820	0.993	0.353–2.798	0.991
Female gender	1.121	0.684–1.836	0.651	1.100	0.745–1.6423	0.633	1.239	0.862–1.781	0.247

*PA* physical activity, *BMI* body mass index, *OR* odds ratio, *CI* confidence interval

## Discussion

The intention of this study is to evaluate the impact of a decreased PA on postoperative recovery after cardiac surgery. Several clinical studies indicate that physical activity offers a kind of protection against the risk of cardiovascular diseases, especially in an older population [1–4, 11–13]. Different factors, such as increased vasodilatory capacity, protection against endothelial dysfunction, increased insulin sensitivity, increased arteriole density and manifest adaptation of stress- and energy-metabolism-related genes have been suggested to explain this beneficial effect [14–17]. Our results indicate that decreased PA pre cardiac surgery is an independent predictive factor for a PCR in patients ≥ 65 year undergoing elective cardiac surgery. Whether or not this is in fact related to the different factors mentioned above, is another question.

In group A, about 37% of the patients are registered as patients with a decreased PA and no significant differences were detected between them and the active patients with respect to the MAE-variables. The percentage of a PCR and MAE-variables in this group is low. This is not surprising, as it is known that the described surgery (isolated CABG, AVR and combined AVR + CABG) can be performed with a low mortality and morbidity, especially if it concerns young patients undergoing elective surgery [18, 19]. On the other hand, we found that the percentage of patients with well-known risk factors is statistically significantly higher in patients with a decreased PA, with exception of a recent myocardial infarction and a history of a neurologic event. That there are significantly more active patients with a recent myocardial infarction probably indicates that active patients are operated on earlier after their infarction than patients with a decreased PA. That there is no statistically significant difference for the variable ‘history of a neurological event’ can be explained by a low incidence of this event (TIA and CVA) in a young population [20]. Logistic regression analysis could not identify a decreased PA as an independent predictor for MAE in these young patients. This is not a surprise because there is no difference in MAE-variables at all between the active and sedentary group. It is understandable that a history of a neurologic event is identified as an independent predictor, because it is a strong predictor for a postoperative neurologic event [20].

In group B, much like in group A, about 38% of the patients had a decreased PA. In contrast to group A, however, this group did show a statistically significant difference in percentage of MAE (*p* = 0.001) between the active and non-active patients. Looking at the different MAE-variables separately, there is a statistically significantly higher percentage of deep wound infections (*p* = 0.001), renal failure (*p* = 0.048), patients with mechanical ventilation > 2 days (*p* = 0.021), ICU stay ≥ 2 days (*p* = 0.001) and hospital stay ≥ 10 days (*p* = 0.001). With regard to the risk factors, and similar to group A, a significantly higher percentage of risk factors was observed in the patients with a decreased PA. A history of a neurological event, recent myocardial infarct and previous cardiac surgery were exceptions. For this group, both renal dysfunction (*p* = 0.001, odds 6.7) and a decreased PA (*p* = 0.003, odds 1.69) are identified as independent predictors for a PCR. Renal dysfunction is, of course, a strong predictor of postoperative renal failure [21].

In group C, 49% of patients were registered as patients with a decreased PA, a subgroup that proves to be significantly associated with a higher risk of PCR (*p* = 0.003). In these patients, the significantly prolonged ICU and hospital stays are responsible for the higher percentage of PCR. Furthermore, compared to active patients, the patients with a decreased PA in this group were more often female (*p* = 0.001), had a higher BMI (*p* = 0.001), had more extracardiac arteriopathy (*p* = 0.001) and more neurological events in their history (*p* = 0.038). Variables predictive of the occurrence of a complicated postoperative recovery were a decreased PA, insulin dependent diabetes and extracardiac arteriopathy.

We recognize several study limitations. First of all, we used the criteria of the CCHP - a self-reporting assessment - to evaluate the patients’ PA status. Roques et al. [9] has been suggested that self-reported PA assessments overestimate true exercise activity and are possibly not specific enough [8, 22]. Tests such as gait speed and hand grip strength have proven to be more specific in older patients [23], but also take up extra time in the work up of the patients. In this case, we needed an easy tool to evaluate patients’ - young and old - normal physical activities over a longer period of time. The CCHP thus represents a user-friendly and less time-consuming assessment for the total group of patients. Leisure-time physical activity, as registered in the CCHP, gives a good idea about what kind of activity peopleperform on a regular basis. Whether or not activity levels 1 and 2 actually correspond to “a sedentary lifestyle” is another question, but we did use this same distinction to identify patients with a decreased PA. This CCHP questionnaire can be used as a first evaluation of PA, and as a selection criteria for further evaluation of patients at risk for a complicated recovery post cardiac surgery.

Secondly, a decreased PA can have various causes, including cardiac, vascular, neurological related pathology, mental disease, frailty et cetera [16, 17, 24]. Another possibility is that patients adopt a decreased PA because they are now ‘patients with a cardiac disease’. In our study we did not distinguish between these different causes. We also realize that a complicated recovery post cardiac surgery does not only depend on preoperative variables, but also on peroperative and early perioperative variables. Even variables such as place of residence and other socio-economical variables can have an impact [23].

Thirdly, while the data are registered prospectively, the study itself is retrospective. It is also a single institution experience, in which isolated CABG, isolated AVR and the combined AVR + CABG have been combined into one group. In addition, coronary artery disease and aortic valve disease may affect the clinical condition differently during the preoperative period.

Fourthly, since the survey was voluntary and not everyone responded, there is some missing data. As already mentioned in previous reports concerning quality of life, this point is frequently ignored but can result in a bias [25, 26]. In this study especially, it is important to realize that the excluded patients were at a higher risk with a higher percentage of PCR. More specific prospective research in the future can include EuroSCORE above a certain value as a risk factor for postoperative events as well as other common variables such as preoperative NYHA class or left ventricular ejection fraction. At least we didn’t perform model discrimination and calibration tests. Either way, the intention of the study was to evaluate whether a decreased PA is an independent preoperative risk factor for a complicated postoperative recovery; not to make a predictive model for it.

To our knowledge, this is the first known study identifying a decreased preoperative PA as an independent preoperative predictor of a PCR after cardiac surgery. While the results of this study may have been somewhat predictable, the presented work now gives proper evidence.

The clinical importance of this study is that, in addition to several co-morbidity risk variables, a decreased PA is a significant independent predictor of a PCR in patients undergoing elective cardiac surgery. As a result, knowledge of this variable is an important factor in evaluating the risk of a complicated postoperative outcome. In addition, we believe it is important for the variable ‘physical activity’ to be registered in cardiac surgery clinical databases, not in the least because the value of PA in a predictive risk model - weighted against other pre-, per-, and perioperative variables - can only be studied if large, multicenter data are available.

Another interesting point in this context is the potential role of prehabilitation to prevent postoperative complications. A well-known example is that of preoperative interventions reducing the incidence of postoperative pulmonary complications as well as, in older patients, the length of hospital stay post cardiac surgery [27]. Of course, there are many more single and combined preadmission interventions to prevent different postoperative complications and the literature can be confusing at times. Here, we refer to two interesting reviews [28, 29]. We also realize, however, that it can be difficult to stimulate patients, waiting for cardiac surgery, to do some preoperative exercise therapy to increase their fitness. Especially since these patients have often just been told not to do physical exercises, because of their cardiac problems.

## Conclusion

Clinicians, cardiologists and cardiac surgeons should be aware that, in addition to several co-morbidity risk variables, a decreased physical activity level is a significant, independent predictor of postoperative complicated outcome in patients aged 65 or older, undergoing elective cardiac surgery.
